# Cycles of (in)visibility: evolvements, constancies, and frictions of menstrual cycles at work

**DOI:** 10.3389/fsoc.2026.1642858

**Published:** 2026-02-27

**Authors:** Samantha Schwickert, Sinje Grenzdörffer

**Affiliations:** 1 Institute for Philosophy and Social Sciences, Sociology of Technology and the Environment, Brandenburg University of Technology Cottbus–Senftenberg, Cottbus, Germany; 2 Institute of Geography, Unit Economic Geography, Bern University, Bern, Switzerland

**Keywords:** critical menstruation studies, menopause, menstrual cycle, menstruation, menstruation and work, work and gender, gender and organization

## Abstract

**Introduction:**

Around half of all working bodies experience a menstrual cycle for a significant part of their (working) lives, followed by (peri-)menopause. Yet, the implications of cyclic embodiment and bodily transitions remain well-concealed, stigmatized, and insufficiently integrated into work organization. Theoretically positioned within critical menstruation research with focus on work organization, our study enhances existing research by offering a *cyclic perspective* which considers the whole menstrual cycle, including changing symptoms over time and the transition to (peri-)menopause. By changing the default linear perspective to a cyclic one, we frame the menstrual cycle as a collective workplace issue rather than an individual inconvenience or problem and advocate for policy reforms. This perspective foregrounds a more holistic understanding of work organization that accounts for diverse and changing body realities.

**Methods:**

For this purpose, we carried out a longitudinal study (2021–2025) to explore and evaluate the effects of the menstrual cycle on the professional lives of women and people with menstrual cycles (WPMC) in Germany. Insights from qualitative interviews and action research workshops during this time period are supplemented by expert perspectives from Germany, Belgium, Ireland and Spain.

**Results:**

The results are organized alongside a differentiation between three spheres (1) individual sphere, (2) organizational sphere, (3) societal sphere. We find that growing awareness, generational shifts, and feminist practices are slowly opening spaces for cycle-sensitive and more caring work cultures, despite ongoing stigma, resistant structural barriers, and anticipated backlashes.

**Discussion:**

Our analysis shows that cyclic practices are largely individualized, which risks burdening WPMC with the additional work of finding solutions for cyclic symptoms within ongoing linear, meritocratic work structures. To counteract these dynamics, this study proposes rethinking workplaces by setting cyclic working bodies as the default. In this way, they are built to accommodate diverse and changing embodied experience by equally valuing care as well as times of regeneration.

## 1 Introduction

Today’s workplaces seem to still be designed around the assumption of a stereotypical masculine, thus, non-bleeding, never changing, predictable body. As Acker’s (1990, 2006) seminal work on gendered organizations has shown, the archetype of the “ideal worker” and related organizational structures are historically shaped around these stereotypical masculine norms. Building on this insight, recent scholarship demonstrates that such organizational structures systematically disregard the physiological realities of the menstrual cycle and menopause, often framing menstruating or menopausal bodies as abnormal, inconvenient, or disruptive (Grandey et al., 2020; Persdotter, 2020; Sang et al., 2021; Steffan and Loretto, 2025).

Many symptoms related to menstruation, such as pain or mood changes, remain underreported or dismissed since menstrual discomfort is often normalized or not taken seriously by healthcare providers (Gaiswinkler et al., 2024; Matteson et al., 2020). Moreover, age-specific and ethnically diverse data are lacking, making it difficult to define what constitutes a “normal” cycle (Matteson et al., 2020; Schultz et al., 2025). Yet, the diverse, cyclic, and changing body realities of women and people with menstrual cycles (WPMC)^1^ are inherent to working environments.

Through the scientific lens of critical menstruation studies, which focus on menstrual health and politics across the life course (Bobel et al., 2020) the topic has gained increasing scientific attention and is now institutionalized as an interdisciplinary research field. Furthermore, menstruating bodies are increasingly considered, integrated and discussed in research on work organization and structures (e.g., Alemu et al., 2025; Fitzgerald, 2013; Furlano, 2025; Grandey et al., 2020; Howe et al., 2023; Levitt and Barnack-Tavlaris, 2020; Sang et al., 2021; Steffan and Loretto, 2025). However, these studies focus mostly on menstruation and/or menopause and do not address cyclic body realities in a more holistic manner, including changes over a lifespan.

The main objective of our article is to position cyclic bodies as the default and to examine how such a reframing would reshape work organization. For this purpose, we explore shifting embodied experiences of the menstrual cycle and transitions to (peri-)menopause in workplace settings in Germany to identify barriers and drivers toward cyclic working environments. Theoretically, our work is situated within critical menstruation studies, with a particular focus on work organization.

The following research questions are addressed: (1) How have discussions and practices around menstruation and cycle awareness in workplaces evolved over the past 4 years? (2) What drives and hinders the transformation of workplaces into cyclic working environments? (3) How could a cyclic perspective reshape work practices and policies toward greater inclusivity and care for the diverse realities of working bodies?

To do so, we examine and evaluate the developments in how the menstrual cycle has affected the working lives of WPMC in Germany over the past 4 years. By framing the menstrual cycle as a collective workplace issue rather than an individual inconvenience or problem, we propose a cyclic perspective that positions cyclic bodies as default and therefore could account for more diverse body realities beyond the focus on menstrual cycles.

Empirically, evidence is gained from a longitudinal study based on 17 semi-structured interviews conducted in Germany between spring 2021 and spring 2025, 7 documented action-workshops carried out in the same time period as well as 4 expert interviews with representatives from Germany, Spain, Ireland and Belgium. Different measures are already being advocated or implemented in these countries. To allow for a coherent research design, our empirical materials are analyzed according to three spheres (individual, organizational, societal), which are deeply interconnected and influence each other in cyclic rhythms.

To address the research questions, we will provide greater details on our conceptual and theoretical framework in the next chapter (part 2). On this basis, we will explain the contextual background and methodological design (part 3). Building on this, we will present our empirical results with a focus on evolvements, constancies, and frictions in the three spheres (part 4), before we discuss drivers and barriers and future visions to overcome the institutional non-acceptance of the menstrual cycle (part 5). From a cyclic perspective and under the assumption of WMPC as the ‘standard working body’, we conclude with theoretical and practical implications for inclusive and careful work organization.

This study highlights that WPMC experience a paradox: they gain more agency about their cycles, yet they still face pressure to conform to stereotypical, linear male-centric productivity standards, leading to symptom concealment and added workloads. The trend of individualized cyclic work intensifies self-management expectations, potentially reinforcing stereotypes and bio-essentialism. To counteract, we advocate for reimagining workplaces that normalize cyclic bodies and value diverse experiences. By promoting a cyclic perspective, the study challenges traditional norms of work organization and emphasizes the need for organizational and societal interventions to support cyclic, menstrual, and menopausal health. This approach offers a more holistic understanding of embodied life in the workplace, emphasizing the full range of bodily realities and proposing cyclicity, change, and lived experiences as key analytical perspectives in organizational theory.

## 2 Conceptual and theoretical framework

### 2.1 Conceptualizing bodies at work

Building on feminist organizational scholarship, this study conceptualizes work organizations as historically structured around assumptions of a stereotypical masculine, linear, rational, and contained body. As Acker’s (1990, 2006) theory of gendered organizations demonstrates, organizational norms, temporalities, and expectations of the “ideal worker” are not gender neutral. Rather, they are implicitly aligned with bodies imagined as stable, predictable, continuously available, and detached from most cyclic bodily processes. While some more gender-neutral cyclic processes, such as sleeping and eating, are more or less tolerated in work organization, feminist and queer studies of work organizations have shown that bodies which further deviate from the “ideal worker” norm, such as reproductive, aging, or “leaky” bodies, are rendered problematic within organizational contexts. These studies highlight how bodily processes that are gendered, cyclic, changing, or difficult to contain, such as menopause (Jack et al., 2019; Steffan and Loretto, 2025), menstruation (Grandey et al., 2020; Sang et al., 2021), or breast feeding (Van Amsterdam, 2015), fundamentally challenge dominant organizational logics grounded in reliability, control, and productivity. Importantly, the studies highlight that rather than prompting structural organizational change, such embodied deviations are often individualized and managed through concealment, self-discipline, and heightened self-responsibility.

Extending this critique, Fuchs (2018) and Pawlett Jackson (2024) explicitly theorize the temporal mismatch between linear time conceptualizations and embodied cycles. Both argue that prevailing living and working rhythms are built on linear, future-oriented, and accumulative notions of time that systematically marginalize cyclic, changing, and regenerative temporalities. Pawlett Jackson (2024) further emphasizes the menstrual cycle as a specific form of embodiment that stands in marked contrast to linear modes of work organization. From this perspective, cyclic bodies are not merely misfits within organizations but actively expose the temporal assumptions underpinning organizational order.

Drawing on this body of work, we conceptualize organizations as environments in which linear organizational temporalities intersect and often clash with non-linear, embodied rhythms. We focus on the menstrual cycle as a cyclic process that affects nearly half of the working population over a substantial part of their working lives, and thus as a paradigmatic yet under-researched example of cyclic embodiment at work. This allows us to examine how organizational structures, norms, and practices engage with bodily processes that are inherently cyclic, changing, and relational.

### 2.2 The menstrual cycle: beyond menstruation

A significant amount of the (global) working bodies are dealing with cyclic and changing body realities in terms of having a menstrual cycle and (peri-)menopausal nonlinearities. Yet, some phases of the menstrual cycle, i.e., menstruation, premenstrual symptoms (PMS), and menopause are highly stigmatized and tabooed especially in workplaces (Grandey et al., 2020; King, 2020; Steffan and Loretto, 2025). In consequence, current workplace arrangements incentivize WPMC to downplay and hide the effects of a cyclic body to avoid being seen as unprofessional (Grandey et al., 2020; Przybylo and Fahs, 2020; Sang et al., 2021; Steffan and Loretto, 2025; Young, 2005). The necessity of concealing menstruation, PMS, or menopause confronts WPMC with additional workload and stress, i.e., in terms of painful bodies, concealing symptoms, presentism, ongoing stigma, lack in access to facilities and balancing workload while experiencing diverse symptoms (Amacker et al., 2024; Gaiswinkler et al., 2024; Grandey et al., 2020; Owen, 2024; Sang et al., 2021). Menstruation and PMS are mostly conceptualized as a problematic ‘female’ experience or challenge that must be individually managed and at all costs concealed by concerned WPMC (Bauer, 2022; Bobel and Fahs, 2020; King, 2025; Persdotter, 2020).

Evidently, the embodied experience of the menstrual cycle is not limited to menstruation or PMS. Nevertheless, most existing empirical studies focus solely on phases of the cycle that are framed as (potentially) problematic, such as menstruation (e.g., Amacker et al., 2024; Gaiswinkler et al., 2024; Schoep et al., 2019), PMS (e.g., King, 2020), menopause (e.g., Steffan and Loretto, 2025), or combinations of menstruation, menopause, or menstrual disorders (e.g., Howe et al., 2023; Sang et al., 2021). While some studies explicitly question the social construction of menstruation, PMS, or menopause as problematic, many others implicitly reinforce this framing. This is most evident in the titles, which associate these phases with deficits, e.g., in Schoep et al. (2019) “Productivity loss due to menstruation-related symptoms” or in Amacker et al. (2024) “Menstrual problems at the workplace^2^.”

As a result of this problem-focused orientation, menstruation and menopause have become the most widely scientifically discussed aspects of the menstrual cycle, while the broader cyclic experience remains largely overlooked. This narrow focus also obscures the fact that symptoms vary not only across cycles but also change over the lifespan.

At the same time, in public discourse cycle awareness, symptom tracking, and cyclic work have become trends many WPMC follow (Andelsman, 2022; Hajkova and Doyle, 2024; Pfender et al., 2025). Guidebooks, newsletter articles, supplement advertising and cycle coaches all promise to provide guidance on using the cycle as a ‘female superpower’ for a successful (work) life. Although these initiatives often include educational elements about menstrual health, the recommendations they make are usually not based on scientific evidence, since scientific research on cyclic work is not yet well developed.

To address this, we suggest a more encompassing approach for organizational theory as well as for critical menstruation studies; one that attends to diverse, cyclic, and changing embodied experiences. Based on this premise, the following section introduces a cyclic perspective on work organization.

### 2.3 A cyclic perspective on work organization

Building on these insights, we propose a cyclic perspective that moves beyond analyzing individual symptoms in isolated phases of the menstrual cycle or focusing solely on stigma or stereotyping. Instead, we advance an analytical lens that conceptualizes cyclic bodies as the default and understands the menstrual cycle, as well as peri-menopausal nonlinearities, as integral dimensions of cyclic, embodied, collective experiences.

**Figure 1:**
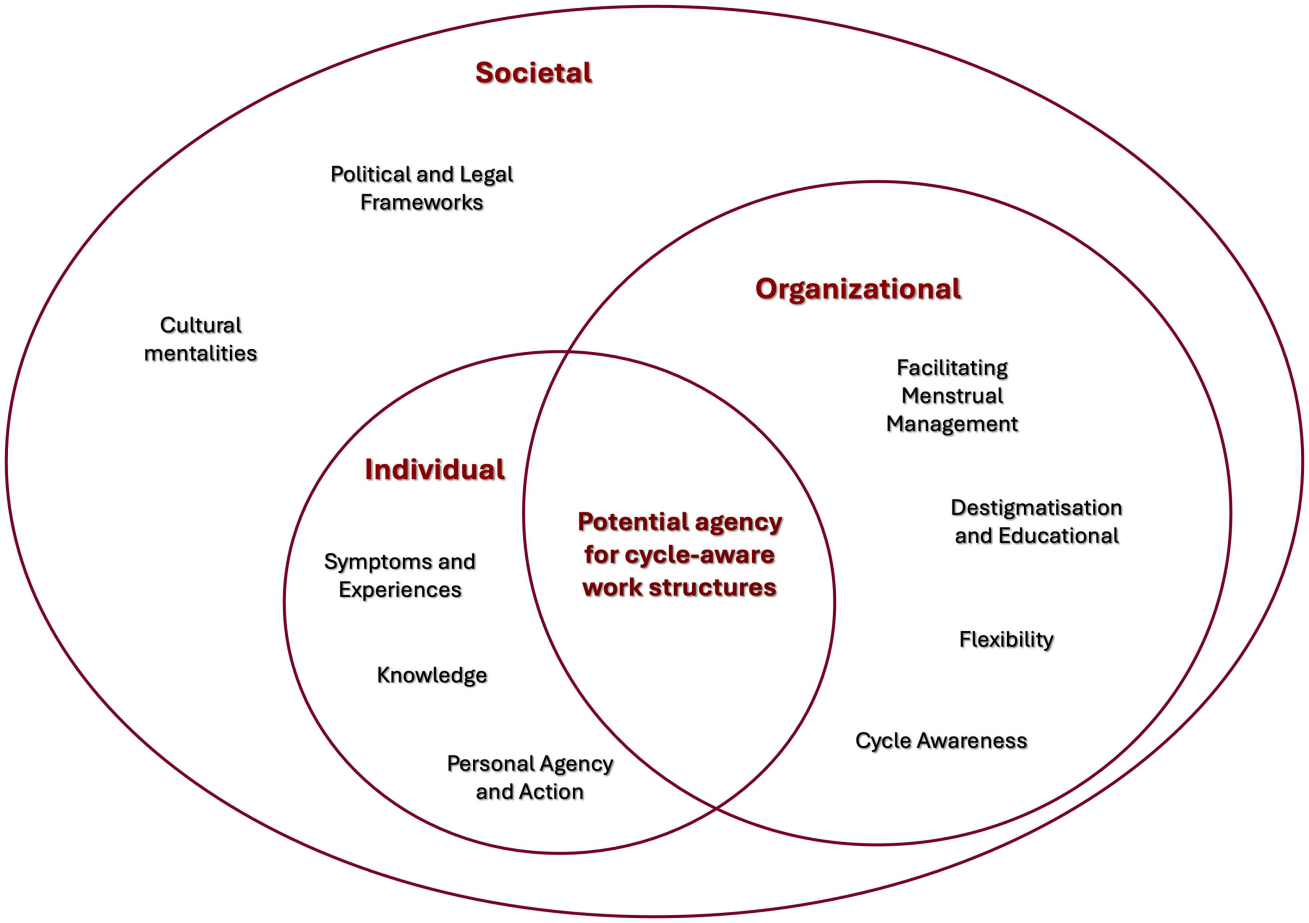
Conceptualizing the menstrual cycle at work: the figure presents the study’s conceptual framework, depicting the menstrual cycle at work as an embodied, cyclic phenomenon shaped across three interconnected spheres: the individual, organizational, and societal.

To ensure a coherent research design, our research project puts cyclic temporalities and bodies of working WMPC in the focus of attention. With a cyclic perspective both in theoretical and methodological terms, we trace the non-linearities associated to a cyclic working body in dominantly linear structures. In resonance with common cross-disciplinary categorisations like “micro-meso-macro” levels of analysis (Dopfer et al., 2004; Serpa and Ferreira, 2019); or multi-level perspectives (e.g., Geels, 2010; Keller et al., 2022), we recognize that embodies realties of the menstrual cycle are influenced by different spheres: 1. The Individual Sphere covering personal experiences, knowledge and agency in reaction to individual body cycles, 2. The Organizational Sphere covering work structures, cultures, and management in reaction to cyclic working bodies, 3. The Societal Sphere covering public discourses, scientific developments and political agendas in view of a cyclic perspective on working environments (see Figure 1). Yet, we saw that the divide between ‘private life’ and ‘professional life’ played a decisive role in our data, which is why we chose to represent the organizational (work) sphere at the same level as the individual sphere. Nevertheless, the individual sphere remains at a micro-level in terms of limited impact. To capture this, we illustrated the organizational sphere with a larger circle that partly includes the individual sphere. From a cyclic view, these three spheres are understood as permeable cyclic loops that transform over time within one sphere, but that are simultaneously inherently interconnected and have a mutual influence on each other.

## 3 Methodological design

### 3.1 Contextual background

In line with research on menstruation as a global topic (e.g., Bhandal, 2020; Furlano, 2025; Gaybor and Harcourt, 2022; Khorsand et al., 2023; Standing et al., 2024), we see a high necessity to research the topic from an intersectional and context-sensitive perspective. In terms of geographical focus, a global-scale review of the policies, guidelines and practices regarding menstruation, menstrual disorders and menopause at work, conducted by Howe et al. (2023), reveals that most studies were conducted in the UK, followed by Australia and the US. It is noteworthy that most EU countries are not high-ranking and that Germany is only included in multi-country articles despite its prominent position in the EU.

While there might be the prejudicial assumption that menstrual stigma is only an ongoing issue in countries in the Global South, Winkler et al. (2024) and Grandey et al. (2020) demonstrate that it remains a crucially under-addressed issue in countries in the Global North, too. A recent empirical study by Forsa in Germany (2025) confirmed this impression. Of the 1,002 participants, 85% thought that menstruation was discussed too little or not at all in public, and 91% thought that there was too little information or education about menstrual cycle health (Forsa, 2025). Complementing these findings, Bauer (2025) situates menstruation as a central site of feminist body politics. While the public discourse increasingly invokes notions of empowerment and de-tabooing, she underscores the persistence of historical patterns of pathologization, naturalization, and gendering in Germany.

From a policy perspective, broader institutional measures addressing the menstrual cycle in the workplace remain rare across the EU despite the high prevalence of menstrual and menopausal symptoms and the considerable number of people affected (Schultz et al., 2025). Exceptions include Spain’s introduction of a menstrual leave law in 2023 (ibid.), a trade union-led campaign in Ireland advocating for menstrual and menopausal workplace policies (Forsa, 2023) and an internal organizational policies of the Left Party in Brussels addressing menstrual health (The Left, 2024).

Taking into consideration our advantage of local knowledge and cultural expertise as two white, cis-female researchers from Germany, we saw this as an opportunity to enhance existing research through in-depth qualitative insights in the German context combined with expert perspectives on an EU level. Our methodological approaches and the specific sample are detailed below.

### 3.2 Methodological approaches

In coherency with the overall cyclic research design, the study employs a longitudinal qualitative approach to explore how cyclic realities can be integrated into work organization. Under consideration of the changing circumstances and embodied realities, we aim to do justice to non-linear temporalities (see, e.g., Fuchs, 2018; Pawlett Jackson, 2024) by talking to the same people in two different moments in time. Within the time of four years from 2021 to 2025, this allowed us to build on the sample indicated in Table 1. The time period mostly evolved due to the life cycles and loops of both the authors and the research participants.

**Table 1:** Research methods, participants and time.

Format	Participants	Number of participants	Date
Qualitative interviews (Phase 1)	Working cis-women in different working environments in Germany (Start-Ups, Medium-Sized-Companies, Corporations)	10	Spring 2021
Action research workshops	Working WPMC at Women’s Barcamp Saarland Germany	Approx.^a^ 10	Fall 2021
	Male and female researchers at CAU Kiel Scales of Transformation Cluster Germany	Approx. 15	Winter 2021
	Students and working WPMC within Yooweedoo Academy Germany	Approx. 90 in total	3 times 2022 and 2023
	Students and working WPMC at Viva con Agua Network-Meeting Germany	10	Summer 2022
	Students and working WPMC at “Lernwerkstatt Erziehungswissenschaften” in Germany	15	Fall 2023
	Male and female people working in organization development in Germany	15	Fall 2023
Qualitative interviews (Phase 2)	Working cis-women in different working environments in Germany	7/10 from Phase 1	Winter 2024/2025
Expert interviews	With four experts from Germany, Belgium, Ireland, Spain	4	Winter 2024/2025
Final action research workshop	Discussion of results of the study with six researchers with expertise in Gender or feminist Studies from University of Bern Switzerland	6	Spring 2025

a Due to the online workshop format, it was not always possible to track precisely how many participants were present for the entire duration.

Our analytical approach combined deductive and inductive elements and proceeded iteratively, guided by the principles of qualitative content analysis (Kuckartz, 2018). We applied this approach to both our interview data and the materials generated in the action research workshops. Across the three rounds of interviews conducted over the four-year research period, we used semi-structured interview guidelines that were adapted to the respective focus and stage of the project while maintaining conceptual continuity. All interviews were transcribed verbatim. The workshops were documented through researcher memos. In the final workshop, participants themselves contributed written reflections on posters, which were photographed and analyzed. Following the coding procedures proposed by Kuckartz (2018) we conducted several cycles of coding, starting with deductive developed codes and added inductive ones to refine our category system.

Deductively our data analysis as well as the semi-structured interview guidelines are organized according to the three spheres: 1. The Individual Sphere, 2. The Organizational Sphere, 3. The Societal Sphere (see Figure 1).

Inductively, in all the spheres three overarching analytical categories emerged—evolvements, constancies, and frictions—capturing different dynamics of change, stability, and tension over time. These categories structured the subsequent presentation of our results and reflect our cyclic understanding of social and organizational processes.

Due to the sensitivity of the topic, we chose to focus on German speaking participants to be able to create a safer space and to react to cultural nuances. Participants were recruited through email lists, LinkedIn, and direct outreach. Ethical standards, including informed consent and confidentiality, were strictly observed given the sensitivity of the topic. Expert interviews were conducted with voices from Germany as well as from Ireland, Belgium, and Spain to enrich the data from Germany with a broader EU perspective. The experts from Ireland, Belgium, and Spain were identified following their participation in the ETUI Conference panel “Integrating Gender into Occupational Health and Safety,” which brought together perspectives on menstrual and menopausal health from different European contexts. Building on this exchange, we selected available participants representing distinct types of expertise: From Ireland, a key staff member of a union campaign tackling menstrual stigma contributed a trade union perspective, highlighting the role of organized labor in advancing menstrual inclusivity at work. The Belgian case serves as a potential good practice example for implementing internal organizational policies on menstrual health, represented by an interview with a leading official. In Spain, an union representative involved in promoting Spain’s menstrual leave law provided insights into legislative frameworks supporting menstrual health. Additionally, an independent Diversity Manager and Cycle Coach for companies from Germany was interviewed. Citations of expert interviews follow the format E.B, E.S, E.I, and E.G, with letters indicating the respective country (Belgium, Spain, Ireland, Germany).

Regarding the longitudinal study, Phase 1 aimed to establish a baseline understanding of personal experiences, cycle awareness at work, and organizational perspectives. Prior to developing the interview guide, a comprehensive literature review was undertaken to build the conceptual framework for integrating menstrual awareness into work structures (Figure 1). Building on the insights from the initial interviews, the study transitioned into a Phase 2 with a focus on transformation. This transformational perspective aims to identify shifts in personal strategies, organizational practices, and public discourses surrounding menstrual health and cyclic work. By revisiting the participants, the study does not only capture individual and organizational transformations but also highlights persistent gaps and opportunities for action.

Finally, the study incorporates action research elements through participatory formats organized over the past 4 years in Germany. These include workshops and collaborative exchanges focused on menstrual cycle awareness in professional settings. These participatory initiatives serve as platforms for identifying challenges, fostering dialogue, and co-creating practical recommendations to improve menstrual inclusivity at work.

## 4 Evolvements, constancies, and frictions—beyond linearities

In response to the first research question, we analyzed our data materials regarding spheres (1) individual, (2) organizational and (3) societal and in these *evolvements*, *constancies*, and *frictions* over the past 4 years. By *evolvements* we understand changes over time, whereas *constancies* capture aspects that remained stable. The term *frictions* denotes constrained or contested changes as well as inner contradictions and ambiguities.

For the individual sphere (3.1) we mainly drew on insights from the interviews in Germany and the action research workshops. For the organizational (3.2) and societal (3.3) spheres, we embedded these insights in a larger national and European context based all gathered data.

### 4.1 Individual sphere

The experience of the interviewees in the individual sphere is characterized by (1) the *evolving* nature of menstrual and menopausal conditions with mostly worsening symptoms over time, (2) *increased* awareness of cycle- or (peri-)menopausal-related needs, and (3) the *constant* negotiation between personal wellbeing and professional obligations.

Our empirical results show that individual behaviors of participants with minimal symptoms or even reduced symptoms due to menopause remained constant over the years. This implies that unless there is a significant physical impact, awareness of the menstrual cycle may not automatically lead to increased engagement and consequential actions with regard to the topic.

In contrast, participants with *worsening* symptoms due to aging or the transition to (peri-)menopause reported *investing more time* in tracking symptoms, refining their diaries, reorganizing non-work activities, or experimenting with medication to sustain or improve their work performance. Some of them have begun to think about aligning their work patterns with their cycles or (peri-)menopausal needs. Nevertheless, such practices were still tentative and provisional. They were not at all formalized or supported by organizational structures and remained mostly self-initiated, inconsistent and rather by chance than actually planned. Additionally, participants did not conceptualize the time they invest in the individually managed practices as additional work but rather considered it an integral part of their everyday practice. Consequently, this increase in mental workload remained concealed at their workplaces.

Menstrual or menopausal symptoms were often managed under the radar of a continuous professional performance to avoid the perception of weakness. Four interviewees revealed to push through work despite severe discomfort, later reflecting critically on this behavior.

*“I was constantly taking way too many painkillers — which isn’t good, of course. But sometimes I just thought, well, you can’t call in sick every time.”* (I.4)

The *friction* between workplace expectations and physical and psychological needs often led to internal conflicts and feelings of guilt or inadequacy. Some interviewees tend to play down their symptoms or emphasize that their employer is not doing that badly when it comes to gender equality. Simultaneously, some participants voiced a fear of overemphasis and re-stigmatization, if the topic was addressed. Concerns also included the risk of being stereotyped based on cycle phases, or being *“mansplained”* (I.4) about one’s own body, which further reinforced hesitation to speak up. Menstrual needs were persistently viewed as private concerns, disconnected from the organizational sphere. Conversely, it is acknowledged, that work is a factor which influences symptoms, as one interviewee put it:

*“It still kind of feels like my own private, personal matter […] And of course, that’s absurd, because my PMS is clearly much worse when I’m stressed. […] I’ve never spent a day in bed because of PMS while on vacation […] So work is very directly linked to my symptoms. But yeah, I’ve never really made a big deal out of it.” (I.6)*

However, the menstrual cycle was not perceived solely in negative terms. Some participants expressed a preference for working differently, i.e., more quietly and independently or from home during menstruation. Some participants also reported experiencing increased energy or creativity during certain phases of the cycle other than menstruation:

*“I can’t really do it in a systematic way, but I do use my cycle tracker on Clue, and sometimes I notice — oh wow, I’ve got a big presentation coming up, and it’s right around ovulation — perfect!”* (I.6)

The findings emphasize the dynamic and evolving nature of menstrual and menopausal symptoms and related needs. The transition to (peri-)menopause, in particular, was described either as “a profound disruption” (I.8) or as a “*liberation*” (I.3). Changes in agency and action appear to be largely symptom-driven and encouraged by a growing focus on one’s own menstrual or (peri-)menopausal health partly influenced by evolvements in the societal sphere (see chapter 3.3). Nevertheless, evolvements in symptoms and in agency and action were managed individually and mostly remained concealed. First tentative attempts to reconcile work obligations with personal wellbeing remained provisional, imposing additional demands of time, effort, and mental load on the concerned WPMC.

### 4.2 Organizational sphere

The organizational sphere reflects the degree to which menstrual or menopausal health and cycle awareness are acknowledged and addressed through formal work structures, policies, and culture. Based on insights from the growing number of interdisciplinary studies on menstruation (see chapter 2.1), popular science literature (Clue App, 2024; Föhr, 2020; Melican and Mountford, 2017; Wiebe, 2020) and the empirical data collected, we identify four interrelated areas of organizational intervention aimed at closing the gap between current work structures and the lived realities of the menstrual cycle. These areas serve as a framework to systematize the wide range of possible measures and their respective focus from a cyclic perspective.

First, facilitating menstrual/menopausal management encompasses low-threshold, practical adjustments that can improve physical and psychological comfort and preparedness during times of PMS, menstruation or menopause in the workplace. Second, destigmatization and education focus on challenging persistent taboos and increasing awareness and knowledge around menstruation, the menstrual cycle and menopause. Third, flexible workplace structures and work-time autonomy can enable WPMC to self-manage potential fluctuations in energy, mood, or physical and psychological wellbeing more effectively. While these measures do not automatically include a structural cyclic work organization, they can contribute toward its establishment in an accidental or individual managed way. Finally, integrating cycle awareness into work structures explores the potential of aligning work tasks and rhythms with cyclic body realities. These four areas should not be seen as isolated or hierarchical but rather as complementary and potentially synergistic approaches toward a more inclusive cyclic organizational culture.

Regarding the facilitating the management of menstruation or menopause, half of the interviewees have access to menstrual products provided by the companies. However, the German expert reports that the provision of menstrual products encounters resistance. Often this is due to logistical complications or lack of initiative, or subcontractors who refuse to handle these supplies, which leads to *frictions* between the needs of WMPCs and company managers. Thus, cyclic health is often not only seen as dispensable or idealistic, but also as something which could be exhausting to push. In this regard, one interviewee had considered asking the department in charge for such tasks for providing menstrual products. However, she ultimately decided against it, assuming women “manage just fine with their own toiletries” (I.3) and feeling hesitant to create additional work for the department. Such perspectives reflect the ongoing framing of menstruation as a private, individual matter rather than an organizational collective responsibility. As the German expert points out, this stands in contrast to examples like, the provision of toilet paper which is a necessity for all working bodies. Notably, none of the interviewees reported structural offerings such as hot water bottles, fans or institutionalized recovery places, even though such measures were often described as supportive when working remotely from home. These examples show that working bodies are per default assumed to do not have cycle related symptoms, which makes the provision of cycle related products optional rather than obligatory.

Regarding destigmatization and education, the findings were mixed. Many interviewees reported that open conversations around menstruation have *evolved*. Yet, several participants described *constant* discomfort or a lack of response when menstruation- or menopausal-related needs were expressed. Gender dynamics played a clear role, with WPMC were more likely to share openly among themselves, while cross-gender dialogue remained highly divided feedback. The reaction of men, when (seldomly) addressed, was ranging from very reserved to very supportive feedback. A generational divide also emerged: younger employees seemed to be more willing to raise the issue and seemed to be generally more open to discuss menstruation and cycle at work. However, older women in leadership were perceived as more able to demand changes since they potentially had another standing and more power than younger women. For example, one interviewee described a situation in which openness about menopause symptoms was expressed, even in a male-dominated space:

*“for example, my female executive sitting next to me just goes, 'I hate this, hot flash, I hate being 50,' and she says that in a room full of men. That’s great.”* (I.9)

In all the cases, an open communication culture about menstrual cycle- or menopausal-related issues was perceived as a beneficial practice that fosters mutual understanding and a sense of care within the team. Particularly, we observed a positive link between an open and supportive team culture and the ability of WMCP to navigate exhausting phases, which helped alleviate stress and mitigate symptoms. In contrast, when it is not possible to openly communicate about such issues, people affected are confronted with different challenges:

*“[…] There’s just no standard way of talking about it yet. One person might say, 'Yeah, my cold is better,' but I’m not going to tell everyone, 'Yeah, my PMS has passed.' That’s just not something that rolls off the tongue as easily as other things.”* (I.6)

All experts emphasized that menstruation remains a highly concealed, tabooed and stigmatized topic in workplaces, even among those who discuss it openly within their families. This aligns with evidence from Spain’s menstrual leave law, where many women reportedly hesitate to use their rights for fear of negative workplace consequences, indicating that destigmatization remains limited (E.S).

The *constancy* of stigma results in low visibility and perceived irrelevance of menstrual health in organizational contexts, reflected in the absence of employee complaints, limited information, and scarce research on prevalence and effective measures.

Flexibility in work time and location was widely perceived as a supportive and enabling factor. While most interviewees already had access to flexible work arrangements in 2021, a comparison of both interview rounds suggests an *evolvement*: adapting working rhythms in response to menstruation or menopausal symptoms appears to have become more normalized. In the earlier interviews, participants often struggled to even imagine utilizing such measures for menstrual-related needs. In contrast, in the second round, many interviewees described actively using home office options or adjusted hours to manage symptoms related to menstruation, PMS or menopause—a notable increase compared to 4 years prior.^3^ Additionally, participants emphasized that it would be helpful to reframe sick leave toward broader understandings of being unable to work independent from the specific reason. In most organizations in Germany one can call in sick without a doctor’s note for a period of 3 days (further discussed in chapter 3.3). Yet WMPC employees still tend to hesitate to take advantage of such rights due to fears of stigma or appearing weak. As presented above in the findings from the individual sphere, several interviewees had feelings of guilt and inadequacy if work could not be done due to cycle related symptoms. Hence, despite the possibility of flexible and autonomous arrangements, the results indicate that it is not fully possible to make use of it without psychological stress. This shows how legal and structural possibilities are entangled with an internalization of sociocultural values and stigmata, which causes *frictions* toward cyclic work environments.

Given the insights from our data materials, general cycle awareness and its organizational integration still seem to be uncommon, which underlines the *constancy* of non-cyclic working bodies. Across both rounds of interviews, the workshops and the expert contributions, a recurring pattern emerges: while awareness and conversation around menstruation have increased, concrete organizational change remains limited. Only one case in the dataset featured a cycle-informed team structure, in which they have started to integrate cycle awareness into planning and task distribution. This included adjusting meeting intensity based on team energy levels and normalizing rest during menstruation. While rare and still in the testing phase, this case presents a promising model for rethinking organizational rhythms and highlights the potential of cyclic leadership.

Nevertheless, as one interviewee emphasized, starting with irony:

“Sure, it’s nice — now you’re allowed to show up with your cycle — but the world of work isn’t made for us. […] It’s simply made for men. It’s just not about us […]. Not at any point. That’s just the f***ing reality.” (I.2)

This experience of gendered discrimination is further underlined by another interviewee who reported a “reversed” logic of men expressing feelings of exclusion or discrimination if topics like menstruation or menopause are addressed:

*“Then there are always people — men, specifically — who ask, 'So what is being done for men?”* (I.3)

Furthermore, a lack of institutional awareness or priority for the topic appears to be a strong *constancy*. Most interviewees reported little to no change within their workplaces regarding structural menstrual or menopausal health support. Reported initiatives often relied on individual engagement rather than structural action. For example, one participant noted that she had added the topic to a meeting agenda, but since she was unable to attend, it was not taken up—highlighting how such initiatives tend to depend on personal continuity rather than structural commitment.

In sum, the organizational sphere reveals a decisive gap between increased discourse on the one hand and meaningful transformation on the other hand. Most of the reported measures often remained symbolic and were rarely integrated into long-term structures. This shows how strong a non-cyclic working body is established and structurally advantaged. From a cyclic perspective, this underscores the necessity for cyclic health as organizational strategy rather than an optional or temporary diversity topic or a private issue of WPMC.

### 4.3 Societal sphere

Resulting from the perception of the participants two different areas of societal change were identified as important: (1) cultural mentalities and (2) political and legal frameworks.

Regarding Cultural Mentalities, our data reveal a complex and ambivalent picture of cultural transformations regarding menstruation and menstrual and menopausal health. Participants observed an *evolvement* in terms of a generational shift and growing societal awareness, with increased emphasis on female health, mental wellbeing, and intersectional feminism. Nearly all interviewees agreed that the public discourse has changed in a progressive and supportive way, and that media coverage of menstrual and menopausal related issues and cyclic work has much increased in recent years. This indicates a partial breaking of the taboo. For example, one participant shared that she could post on LinkedIn about menstruating “and everyone would just be like, ‘Cool’ — like it” (I.2). Moreover, participants observed changes in the discursive tonality, from a suffering-oriented to an empowerment-oriented perspective, particularly in relation to professional life.

In contrast, these transformations are often experienced in *friction* to a broader cultural shift: they are often perceived as incomplete and confined to certain social ‘bubbles’, rather than indicating widespread structural change. Several participants stressed the gap between private awareness and public or structural implementation. As the interviewee, who could post about menstruation on LinkedIn finished: “everyone would just be like, ‘Cool’ — like it. But no one actually organizes their work according to that.” (I.2).

Additionally, a further *friction* could be observed in concerns about a growing backlash and political regression in response to increased gender discourse, particularly in connection with far-right movements. Signs of misogyny and antifeminist narratives were described as gaining momentum in certain cultural and political areas, causing feelings of threat. The German, Belgium, and Irish experts echoed these concerns about a general shift to the right in Europe, which is often associated with transphobia, misogyny, and threat to WPMC’s rights.

Regarding political and legal frameworks, participants agreed that the legal debates around menstrual health are *constantly* rare, although they are increasingly present in political and organizational discussions. Regarding the European landscape, our data material reveals the following picture:

While an initiative for an EU menstrual leave policy was launched by the Italian parliament in 2017, the proposed directive was not granted (European Parliament, 2021). Thus, it remains in the hands of the nation states to offer a legal framework that supports WPMC in this regard. Regarding the countries of the experts, the interviews revealed the following insights:

Germany’s legal framework does not include menstrual leave. However, one could argue German’s policy is still beneficial for people affected, since typically, no doctor’s note is required for up to 3 days of sick leave (§ 5 EntgFG). Hence, most people suffering from menstrual or menopausal issues could take time off without needing a doctor’s note or diagnosis at all. Yet, companies can demand certification from the first day (§ 5 EntgFG) — potentially creating barriers for those affected. The German expert summarized the legal situation on menstrual leave in Germany as follows:

*“Legally, I don't think we need it [Menstrual Leave Law]. But it would have the major benefit of breaking the taboo, distinguishing between illness and menstrual needs. It could raise awareness and help people take their own symptoms seriously. It’s less about regulation and more about starting a cultural shift.”* (E.G)

To date, Belgium and Ireland are also lacking specific national legislation on menstrual leave, and the implementation remains voluntary or non-existent in most workplaces. In contrast, Spain currently serves as a landmark case. The country has become the first in the EU to introduce national legislation on menstrual leave, which allows time off for people experiencing severe menstrual pain:

*“This law will help ensure that menstrual pain is no longer normalized and that it starts to be treated with the importance it deserves. Menstruation will no longer be a taboo that stigmatizes women.”* (E.S)

Yet, the actual availment remains low: only 14% of women had applied for menstrual leave (Sociedad Española de Contracepción (SEC), 2024). According to a national survey from Spain, over half of women fear negative workplace consequences, especially younger women and those with lower education or income levels (E.S). This highlights the continued stigma and fear of discrimination that can undermine implemented polices. Additionally, the Spanish expert criticizes the fact that women need a previous diagnosis from a doctor, which is a major barrier to WMPC applying the law. Despite these concerns, Spain’s law has sparked some international attention and inspired comparative reflections across Europe. The Irish expert noted that their union’s campaign received much more media coverage because the discourse was already underway.

Nevertheless, policy discussions remain *constantly* limited at an overarching EU level. Interviewees and experts highlighted the lack of consistency in national sick leave regulations and the absence of unified EU standards. Some experts saw potentials in aligning menstrual leave with existing frameworks for parental leave under EU law. However, opinions differ on whether a joint EU legislation would be desirable or effective. Broader debates on cyclic health in the workplace, beyond menstrual leave, are still lacking.

*“We have made such incredible moves in protecting and advancing so many aspects of a person's working life. It's just when it comes to periods, we're just not doing anywhere close to enough. Like, it's just absolutely not there yet.”* (E.I)

In summary, cultural change is underway, but it is shaped by various *frictions*: progressive shifts in public and private discourse coexist with stagnation at the structural level and resistance or even backlashes from conservative forces. The evolving narratives and legal framework around menstruation, especially those that move away from suffering and toward empowerment, signal important steps forward. Yet, they remain fragile, context-dependent and contested.

Overall, the findings across all three spheres reveal an ambiguous and complex landscape. First, we see *evolvements* in individual experiences and personal agency, as well as in awareness and openness in discussing cyclic health at individual, organizational, and societal levels. These developments indicate meaningful shifts in how WPMC navigate and articulate their cyclic experiences. However, despite these evolvements, corresponding structural changes within workplaces remain limited. In terms of *constancies*, our results indicate that organizational transformations regarding cyclic health remain fragmented and unreliable, and many work structures continue to be shaped by linear temporalities that fail to accommodate cyclic embodiments. Thus, while discourse and individual practices may be shifting, institutional arrangements stay largely unchanged. The misalignment between evolvements and structural constancies produces significant *frictions*. WPMC who seek to protect their menstrual or menopausal wellbeing at work often bear the burden of managing it alone, investing additional time, money, and emotional energy. Cyclic health thereby remains framed as an individual issue affecting a “minority group,” rather than as a collective workplace concern relevant to all bodies. Further *frictions* become visible in policy developments: advances such as national menstrual leave laws (e.g., Spain) serve as important reference points, yet low uptake, implementation gaps, persistent stigma, and political resistance continue to constrain meaningful structural change.

Taken together, these *evolvements*, *constancies*, and *frictions* raise the crucial question of what drives or hinders workplace transformation and how workplaces might be reimagined if cyclic bodies were positioned as the norm.

## 5 Barriers, drivers and future visions for cyclic bodies as default in work organization

While the results section presented evolvements, constancies, and frictions within the individual, organizational, and societal spheres, now, we synthesize these findings into broader, cross-spheres mechanisms. To answer our second and third research question, we discuss key barriers and drivers that shape the transformation toward cyclic health at the workplace. In a second step, we assume a cyclic perspective that sets cyclic bodies as the norm to discuss future visions and practical implications based on identified barriers and drivers.

### 5.1 Barriers

#### 5.1.1 Stereotypical male as norm

Previous research showed that the workplace remains structurally oriented toward a stereotypical male default, which is linear, predictable, and always available for work (Fitzgerald, 2013; Owen, 2024; Steffan and Loretto, 2025). When WPMC individually try to work differently and take their cyclic needs seriously, they partly begin to break with this male default. Thereby, the tension between workplace expectations and cyclic needs often leads to internal conflict and feelings of guilt or inadequacy, making it hard for WPMC to fully do so. Efforts in all three spheres to promote cyclic health are accompanied by fears of re-stigmatization. These fears find echo in the global anti-feminist backlash of the last years (e.g., Gergorić et al., 2025; Möser, 2022; Sanders and Jenkins, 2022) which leaves its traces in questions of cyclic health and work organization. One participant voices her concern as follows:

*“There’s this whole thing with the AfD or Trump-style rhetoric—like, come here, be a real man again, take back control over your woman. […] It feels like two completely separate worlds developing—and I see this as a major danger.”* (I.2)

Furthermore, the rigidity of traditional work models (e.g., 9-to-5 schedules), branch-specific limitations, and reduced or currently reducing funding for diversity initiatives (see also Murray and Bohannon, 2025)^4^ were mentioned as further constraints. As argued by Reid-Musson (2018, p. 893) in the context of working structures of migrants in Canada, “there is a racial, colonial, gender, and sexual politics to rhythms and the differential and deeply illiberal interventions operating at the level of rhythms.” Yet, she also sees the potential of “rhythms creat[ing] opportunities for counter-rhythms, place-making and disruption” (ibid.). Transferred to the context of this study, we can observe a similar dictation of rhythms by prevailing work organization according to an assumed non-cyclic body. Yet, as will be discussed below, we also see potentials for “counter-rhythms” toward inherently cyclic working environments.

#### 5.1.2 Taboo, shame, and stigma may change, but cyclic needs are still concealed and privatized

Our findings correspond with recent research showing that menstruation is increasingly discussed through narratives of destigmatization and empowerment (Bauer, 2025) and that movements challenging menstrual taboos have gained significant traction (Bobel and Fahs, 2020; Gottlieb, 2020). These broader trends are reflected in our data, which indicate a modest increase in individual communication about cyclic symptoms. Yet our results show that discussions mostly remain in the individual sphere and especially happen among cis-female peers within privileged, well-educated circles.

While individual awareness or communication about cyclic symptoms may rise, it does not necessarily translate into personal action or increased visibility of the issue at the organizational level. Correspondingly, our findings in Chapter 3.2 reveal a vicious cycle of communication patterns, knowledge gaps, and stigma. Taboo and shame surrounding menstrual and menopausal symptoms remain deeply embedded in organizational cultures, hindering open discussions about menstruation, cycles, and menopause. Additionally, there is a real or perceived resistance by (mostly) men, who could express feelings of exclusion or discrimination if cyclic needs are addressed. Therefore, integrating cyclic perspectives in workplaces is seen as unjust and exaggerated demand, which creates new forms of menstrual, cyclic, and menopausal stigma. This aligns with insights from recent studies, which indicate that forms of menstrual stigma may vary and evolve over time but generally persist and continue to perpetuate stigma against WPMC (Bauer, 2025; Gottlieb, 2020; Grandey et al., 2020; Olson et al., 2022; Winkler et al., 2024).

#### 5.1.3 Meritocratic presentism

So far, enhanced individual engagement on cyclic body issues do not appear to have turned into actionable strategies for workplace measures. One reason for this might be internalized pressure to perform and the perceived obligation to remain productive under all circumstances. The phenomenon of presentism (Amacker et al., 2024; Schoep et al., 2019) and the pressure to hide menstrual or menopausal symptoms, whether by preventing visible signs like blood leakage or by maintaining high performance despite pain, severe PMS, or brain fog in menopause, is echoed in our findings.

Furthermore, our results suggest that these coping strategies, once internalized, continue to shape behavior even when WPMC begin to critically examine them. This aligns with a previous study on menopause at work (Steffan and Loretto, 2025), showing that although WPMC may recognize menopausal health as an important organizational topic, they often continue managing or concealing symptoms to avoid being perceived as oversharing or deviating from core business agendas. Thereby, this topic is again shifted into the individual responsibility and WMPC experience the pressure to perform a stereotype of an ‘ideal’ worker (Steffan and Loretto, 2025). Our findings suggest that this pattern applies not only to menopausal symptoms but also to menstrual and PMS-related experiences.

#### 5.1.4 Cyclic work as ‘female’ superpower

In recent years, practices of cycle awareness, symptom tracking, and so-called cyclic work have gained popularity among many WPMC, often promoted through guidebooks, newsletters, coaching, and commercial products that frame the menstrual cycle as a “female superpower,” despite the fact that most of these recommendations lack a robust scientific evidence base, as research on cyclic work remains limited (Andelsman, 2022; Della Bianca, 2021; Hajkova and Doyle, 2024; Pfender et al., 2025).

Many participants expressed an interest in the concept of cyclic work and appeared informed about the relevant recommendations, particularly those who experienced symptoms during menstruation and PMS. Some reported feeling less productive and energetic during these phases of their cycle, a pattern also supported by other studies with broader representation (Amacker et al., 2024; Armour et al., 2019; Schoep et al., 2019). However, it remains challenging to determine whether fluctuating productivity is a matter of self-perception or reflects a genuine decline in work output (Grandey et al., 2020; Ronca et al., 2025), making it unclear whether cyclic work would effectively address these issues. In contrast, previous studies found that WPMC encounter additional workloads in the form of both physical and psychological challenges while managing menstruation and menopause at work (Grandey et al., 2020; Owen, 2024; Sang et al., 2021; Steffan and Loretto, 2025). Our results indicate that the trend of ‘cycle awareness’ and ‘cyclic work’ expands this existing extra workload. Beyond hiding symptoms, always being equipped with the right products and medication, and mental load because of stigma and shame around menstruation, WPMC may feel pressured to educate themselves about their cycles, track symptoms, invest in supplements or medication, seek individual cycle or menopausal coaching, and individually reorganize their work accordingly. Thus, a meritocratic work culture toward ‘productive’ cyclic work is fostered. In consequence, there is a risk of overshadowing the stigmatizing structures in the prevailing work organization that run contrary to ‘unproductive” regenerative time and the right to take sick leave if symptoms such as pain are experienced.

Moreover, framing the menstrual cycle as a unique ‘female superpower’ or suggesting that ‘women just need to work differently than men’ risks reinforcing gender stereotypes and biological essentialism. Bauer (2025) argues that menstruation is often constructed as “the most natural thing a woman can experience”, thereby strengthening the association between femininity and nature. Following this line of thought, we argue that narratives portraying the cycle as a ‘female’ superpower risk re-essentializing and re-stereotyping by equating ‘female’ with ‘menstruating’ and ‘cyclic’. Such framings implicitly position ‘female’ and thereby cyclic work as the other to stable, ‘male’ work, thus, reaffirming binary and hierarchical gender logics rather than challenging them.

Reflecting on the paradoxical and often contradictory “*menstrunormativities*” identified by Persdotter (2020), we suggest that the current trend of cycle tracking and cyclic work risks exposing WPMC to even more conflicting norms, extending beyond menstruation to the entire cycle. WPMC are thus confronted with a dual expectation: to either suffer from their cyclic and changing bodies or to transform them into a ‘superpower’. Building on Persdotter’s work (2020), who expressed one example of paradoxical “*menstrunormativities*” as follows:

*“Don’t tell anyone you’re menstruating! But be proud of your functional body! It’s perfectly natural to bleed! That’s gross, conceal!”* (Persdotter, 2020, p. 359)

We propose an equivalent in the form of contradicting *cyclenormativities*:

*During ovulation do that. During menstruation do this. Track your symptoms. Change your diet. But be proud of your natural body. Embrace your mood swings and use them productively. Having menstrual or menopausal symptoms is normal. But if you cannot work because of your symptoms, it is your own fault.*

Analyzing our results over all three spheres shows that the current evolvement of cyclic work is highly individualized. It places responsibility and additional workload onto WPMC while remaining largely concealed. In this way, this approach does not foster inclusive and caring workplaces. Rather, it risks exacerbating neoliberal exploitation, stereotyping, and (bio-)essentialism of WPMC.

Despites these barriers, we could also identify important drivers toward cyclic working environments, which we will detail in the following chapter.

### 5.2 Drivers

In contrast to the described male-default and performance-driven work structures, we identify emerging cultural shifts and new leadership dynamics in all three spheres as key drivers of change.

#### 5.2.1 Cyclic work as diversity topic

Interviewees identified intersections with broader diversity and inclusion initiatives as promising entry points for change. Experts and union representatives linked the issue to occupational health frameworks and legal obligations:

*“Supporting them [employees] to all their needs, which by law, under the health and safety acts, you have to. It's just quite frankly bizarre to me why menstruation is left out of that conversation.”* (E.I)

Beyond inspiration, the role of unions and broad societal organizations was emphasized as a crucial driver to create structural pressure and support national frameworks that individual companies alone cannot achieve. Furthermore, coordinating cyclic policies as collective workplace issues beyond individual body realities could foster more inclusive and diverse working environments beyond visible topics of menstruation, PMS or menopause. When cyclic health is embedded into a wider framework of health and diversity topics, including caregiving, part-time work, parenting, and reproductive or mental health, its relevance becomes more visible and harder to dismiss. Such an intersectional perspective aligns with shifts in work cultures that are becoming more accepting of diverse life models and intersectional realities (see also Byrd and Scott, 2024).

#### 5.2.2 WPMC in powerful positions and as valuable workforce

Crucially, increasing numbers of WPMC in leadership positions were mentioned as essential drivers for change as they are more likely to raise the topic and to create structural visibility. Some participants suggested that menopause receives more recognition than menstruation not because it is less stigmatized but because the WPMC who raise this issue are more powerful. This is in line with results from previous studies (Grandey et al., 2020; Howe et al., 2023). These insights point to the importance of representation, experience, and positional power in shifting workplace norms and cultures: menstrual health appears to become more actionable when voiced by those with institutional authority and personal relevance.

Another central driver mentioned was the growing concern around skilled labor shortages and retaining WPMC in the workforce, particularly those in midlife facing menopausal symptoms. Several interviewees argued that ignoring menstrual and menopausal health risks the long-term productivity and retention of highly trained staff. Hence, failing to act on menstrual and menopausal health was represented as a “waste of human potential”:

*“Big companies will have to act because they're losing a whole workforce of women over 40. And you can’t just fire them — this isn’t even a disease. It’s a natural condition that’s only now being recognized.”* (I.8)

This is in line with a growing number of studies discussing how menopause is contributing to choice and control over women’s future work trajectories (Krekula and Vickerstaff, 2020; Léime et al., 2017; Steffan and Loretto, 2025). Framing menstrual and menopausal health as a workforce retention strategy can be a promising entry point to increase urgency and legitimacy as a cost-efficient measure. This illustrates a rather strategic approach that constructively addresses the challenges of the modern workforce without standing in contrast to neoliberal critiques.

Increasing the representation of WPMC in positions of power and acknowledging their importance as workforce would make it more feasible to normalize cyclic bodies in organizational contexts.

#### 5.2.3 Increased menstrual and menopausal education

To address the persistent lack of comprehensive data and public knowledge, a growing momentum around education and awareness-building was mentioned as an important driver of change. A new generation of WPMC, equipped with greater access to digital knowledge platforms and peer exchange, was seen as particularly impactful in shaping more open conversations around cyclic health and work. This is in line with current research indicating a shift toward destigmatization and empowerment in communication and education around menstruation (Bauer, 2025). An increase in menstrual literacy, particularly in male-dominated fields and medical education, was cited as a major driver to normalization and policy development:

*“And on the other side, for men and non-menstruating people, there needs to be more education — at schools, at universities, in medical studies. I’m always shocked […] I don’t think this is something a company can fix on its own.”* (I.4)

#### 5.2.4 Men as allies and cyclic as well

The support and feminist allyship of male colleagues and leaders were described as a powerful and sometimes surprising resource. Participants experienced that men who had close connections to menstruating partners, children, or friends were often more empathetic and ready to engage, especially when the topic was normalized at home but still silenced at work. Expert voices reinforced the importance of including male leaders and decision-makers in the process to broaden legitimacy and to overcome sector-specific resistance. Questioning the ideal of linear, predictable, and ever-productive bodies would not only benefit WPMC but all workers, as this stereotypical masculine norm fails to reflect human variability which also affects men. Recognizing cyclicality as a default condition could foster more inclusive conceptions of embodiment, given that all bodies are subject to biological, emotional, and psychological rhythms of change (see also Alaimo, 2010; Fuchs, 2018; Grosz, 2004; Reid-Musson, 2018). The role of men was not the focus of this study. However, it warrants further investigation to enhance our understanding of how male allyship can contribute to more inclusive workplaces. Additionally, exploring the inherent cyclicity of cisgender men may provide valuable insights into fostering a more comprehensive approach to workplace inclusivity.

In conclusion, our findings indicate a growing individual, organizational and societal awareness of cyclic health over the past 4 years and a generational shift toward more open discussions of these topics. Despite times of multiple crises, in which gender equality and feminist demands are facing resistance, our findings suggest that there is currently a window of opportunity to drive change toward more inclusive and caring cyclic workplaces.

Together, these developments offer hope for the creation of caring workplace cultures that embrace diverse cyclic body realities.

### 5.3 Cyclic bodies as the norm for future visions

Grounded in the identified barriers and drivers, we want to contribute to traditions of feminist futuring by developing workplace visions from a cyclic perspective. Envisioning alternative futures can help to unlock new practices and ways of thinking to shape more equitable worlds (Coleman and Jungnickel, 2023). Rather than developing scenarios or predictions, the goal of feminist futuring is to imagine and articulate visions and utopias that point toward more just and sustainable futures, and to explore pathways to bring these into being (Gümüsay and Reinecke, 2022; Priyadharshini, 2023). Feminist futuring is often grounded in a non-linear understanding of temporalities (i.e., Baker, 2018; MacLeavy et al., 2021; Reid-Musson et al., 2020). In concert with these perspectives, we propose workplace visions in which the cyclic and ever-changing nature of the body is the norm.

In terms of feminist utopias, a consequential work organization in loop with cyclic bodily changes and the prioritization of personal wellbeing over meritocratic and linear productivity norms can be seen as a “feminist glitch: a vehical of refusal, a strategy of nonperformance” (Russell, 2020). According to Russell (2020), a feminist glitch is the use of error as empowerment, a way to challenge systems of oppression by embracing failure, disruption, and nonconformity as feminist acts. In this understanding, the conceptual framing of cyclic bodies as default working bodies can be perceived as a feminist glitch against patriarchal and neoliberal norms. As an amplification of our “feminist glitch,” we are, hence, discussing future visions on basis of the discussed drivers and barriers.

Our study confirms that the combination of the assumption of a *stereotypical male as workforce norm* and *ongoing menstrual stigma* continues to influence individual behavior, and that resulting individually implemented cyclic work can burden WPMC with an additional workload rather than relieving it. These insights support a future workplace vision beyond individual management and coping in which cyclic body realities can be openly and collectively addressed.

In this vein, when asked about imagined future developments, many participants wanted to enhance body consciousness and to *collectively* increase the implementation of cyclic work. As one interviewee put it:

*“Encouraging women to listen more to their own bodies […] not to say […] I'm going to take pain medication or I'm going to push through it, which I did for years in a really extreme way, but to enter more strongly into this inner dialogue and that this becomes normal.”* (I.4)

Few participants even called for a re-framing or re-interpretation of stigmatized symptoms or cycle-phases, gaining back ownership about such framings and concerned feelings and taking them seriously:

*“What does the saying ‘Are you having your period?’ [said in a teasing way] actually mean? Maybe this saying also has something positive […] Because that turns the dynamic. I feel like [now] some women are able to respond ‘Yeah, thank you […] for noticing that, so really don't talk to me about that stupid topic anymore today and tomorrow.’”* (I.9)

Here we see a twist of normalizing and collectivizing cyclic body realities instead of stigmatizing them as either ‘unchosen misery’ or a ‘superpower’. Today, acting like this is only possible *in powerful positions* including a secure working contract and with a certain amount of self-confidence and/or standing within the team. However, we argue that these two examples represent promising entry points for changing the way the cycle is framed: normalizing cyclic bodies and taking physical and psychological changes seriously instead of dichotomising them. Accordingly, a cyclic perspective implies future workplaces that include regenerative and pausing phases as well a re-interpretation of changing symptoms and cyclic bodies as a habitual recurring part of working lives. In concert, with the identified drivers, *WMPCs in powerful positions* can decisively contribute to this normalization of cyclic working realities.

Grounded in *increased menstrual and menopausal visibility and education*, pragmatic suggestions like the provisions of menstruation products, ensured clean and well-equipped toilets as well as dark-colored furniture and breathable workwear already exists (e.g., Forsa, 2023). Yet an assumed cyclic body norm turns them into an unquestionable necessity rather than an anti-discrimination measure for a minority group. In a similar way, topics of workplace and work-time autonomy and flexibility are already recommended (ibid.). However, a cyclic perspective allows for its possibility and application to *any* bodily concerns avoiding sexist re-stigmatization or discrimination of WPMCs. Additionally, it implies menstrual leave or a broader understanding and acceptance of reasons for taking sick leave.

A cyclic perspective also holds potential for sectors in which workplace and working-time flexibility is limited. By recognizing cyclic bodies as the default, organizations could, in principle, organize shift systems in ways that better align with embodied rhythms rather than assuming stable and linear availability. However, feminist scholars indicate that individualized practices such as cycle tracking and personalized scheduling with smart technology risk reinforcing pressures to manage one’s body productively and efficiently (Andelsman, 2022; Della Bianca, 2021; Hajkova and Doyle, 2024; Pfender et al., 2025). Rather than challenging dominant productivity norms, such approaches may inadvertently reproduce the very organizational logics they seek to transform. At the same time, interpreting this tension as a “feminist glitch” allows for a more nuanced reading of technological interventions. When detached from individualized self-optimization and instead implemented as collective organizational tools, smart technologies may offer opportunities to support adaptive scheduling practices that ensure adequate workforce coverage while prioritizing wellbeing across diverse bodies and genders.

Taking the debate on meritocratic tendencies further, both literature (Andelsman, 2022; Della Bianca, 2021; Hajkova and Doyle, 2024; Pfender et al., 2025) and our empirical insights show how societal trends to use menstrual cycle phases as “*female superpower*” risk falling into a trap of unlimited productivity. In consequence, visions of cyclic working environments invite broader critical reflections on contemporary capitalism and the resulting alienation of humans from nature and ecosystems. Along these lines, we encourage a normalization of organizing work according to one’s wellbeing, influenced by many different cycles and changing bodily needs.

On a more structural level, a cyclic body norm would hold legal and political institutions accountable in a different way. Propositions entail the implementation of menstrual, cycle, and menopausal health as part of the labor law or gender equality legislation, as well as its inclusion in collective bargaining agreements to ensure sector-wide implementation. Based on the insights from our expert interviews, EU-wide implementation is seen as highly desirable to accelerate change, including broader discussions on current linear working conditions. As a final point that goes beyond current focal points in critical menstruation studies (see chapter 2), a cyclic body approach considers not only menstruation, but the whole menstrual cycle and various life stages and cycles. In this vein, *men could enact allyship* by questioning the assumed male body linearity and by challenging the framing of cyclic embodiment as an issue that concerns only menstruating bodies. Men, too, experience cyclic bodily processes, yet they are often caught in pressures to conform to the ideal worker norm to maintain privilege and status.

Consequently, if all bodies are recognized as inherently cyclic and ever-changing by default, work organization can better accommodate diverse lived experiences and support health and wellbeing rather than undermining them. Moreover, cycle awareness can be understood as situating the human cyclic body as one of many interrelated natural cycles that shape and influence human and ecological systems. Or in the words of an interviewee:

*“I’d like a really big cycle board. Where you can just see each other all the time, wherever we are. So always on this … Somehow between eco and ego. So where do we stand in the ecosystem as an organization, as a whole? And where do we stand individually?” (I.2)*

## 6 Conclusion

This study highlights that, despite increased individual and societal awareness and discussion around the menstrual cycle and menopause, organizational transformations toward cyclic working environments remain fragmented. WPMC experience a paradox: While they have gained greater agency regarding their bodies, they still face pressure to conform to stereotypical male-centric productivity standards, leading to ongoing concealment of symptoms and additional workload. Moreover, the upcoming, individualized trend of cyclic work further intensifies self-management expectations. WPMC are encouraged to track symptoms and seek remedies to transform their cyclic experiences into a “female superpower.” However, we identify these emerging *cyclenormativities*, building on Persdotter’s concept of “*menstrunormativities*” (2020), as potentially exacerbating neoliberal meritocracy, stereotyping, and bio-essentialism rather than dismantling them.

To counteract these dynamics, this study proposes rethinking workplaces in ways that take cyclic bodies as the default, value care as well as times of regeneration, and accommodate diverse and changing embodied experiences. By applying a cyclic perspective, we contribute to feminist organizational theory by extending Acker’s (1990, 2006) concept of gendered organizations. Feminist and queer studies of work organization (Grandey et al., 2020; Jack et al., 2019; Sang et al., 2021; Steffan and Loretto, 2025; Van Amsterdam, 2015) have shown that gendered, cyclic, or potentially leaky bodily processes are highly individualized and managed through concealment, self-discipline, and self-responsibility. Building on this extensive work, we used the example of the menstrual cycle to show how cyclic body realities substantially clash with working environments that are commonly organized around a stereotypical linear and unchanging working body. In line with Fuchs (2018) and Pawlett Jackson’s (2024) theorization of cyclic conceptions of time, we foreground the menstrual cycle as a paradigmatic case of cyclic embodiment at work showing how WPMCs bodies expose the linear temporal foundations of organizational order rather than merely deviating from them. Consequently, our analysis demonstrates that work organization is not aligned with gendered, changing and cyclic body realities, and instead remains oriented toward a linear and stable ideal that does not exist in reality.

Theorizing cyclic embodiment as a central yet systematically marginalized dimension of work organizational, we challenge this assumed linearity by setting bodies of WPMC as default. Through this “feminist glitch” we could disclose the existing frictions between the ideal working body and the actual working body realities of WPMCs. However, this analytical perspective also allowed to identify transformation potentials as cornerstone for future cyclic workplace visions.

Empirically, our findings extend the current research on menstruation in work organization by examining the entire menstrual cycle and its changing nature over time. By tracing evolvements, constancies, and frictions across spheres, we reveal how cyclic realities unfold in non-linear and sometimes contradictory ways. Currently, this unfolding incurs costs for WPMC. Therefore, our study calls for practical interventions to shift responsibility for change away from WPMC and toward organizations and society, which can be found in the Appendix.

### 6.1 Limitations

As an exploratory, qualitative, empirical study, this research acknowledges several limitations. All interviewees work in sectors where remote work is easy to implement and already well established, which might not apply to different sectors. Additionally, this study focused on people aged between 20 and 50. Since many WPMC transition into menopause after the age of 50, future research should include broader age ranges. More generally, it must be considered that those willing to participate in such studies or action research workshops may already feel more open, educated and emancipated regarding the still-tabooed nature of the topic. As a result, our findings may reflect perspectives from more progressive or rather feminist, emancipated social groups, introducing a potential bias toward greater openness. Lastly, our study only covers perspectives from well-educated, abled-bodied, mostly white, cis-gender women. Menstrual and menopausal experience are a highly intersectional topic, and further research with more diverse groups is needed to cover different discrimination axes in relation to cyclic bodies.

### 6.2 Further research recommendations

Considering the limitations of our study, further research should evaluate practical measures within different organizational structures and with broader participation, including individuals from different professional settings and various backgrounds. Furthermore, we propose applying our approach of a cyclic perspective in different political and geographical contexts to be able to identify more nuanced conceptions of drivers and barriers toward increased cyclicity on a societal level beyond singular organizations. Furthermore, our empirical insights indicate that the ways of cyclic work organization promoted by blogs, guidebooks, and cycle coaches remains scientifically unexamined, which invites broader research to increase knowledge on cyclic health beyond stereotyped menstrual cycles. Additionally, it would be theoretically interesting to interlink a cyclic body perspective with more encompassing economic models like circular economy, caring/solidarity economy or postgrowth debates. Through a focus on socioecological and mostly concealed costs of a linear economic growth paradigm, they are already challenging linear economic constructions. Setting an interconnected cyclic system both in terms of macroscale perspectives on global economic and in terms of microscale body realities could enhance an overall understanding of the shortcomings and concealments of purely linear thinking and organization and create opportunities for loops of change.

To close the cycle of this paper: Today’s workplaces are still designed around the assumption of a stereotypical masculine, thus, non-cyclic, never-changing, predictable body. Yet, when we start to *acknowledge* diverse and cyclic body realities *as default*, this does not need to be the future.

## Data Availability

Due to the sensitive nature of the qualitative data, the raw data are not publicly available. Anonymized excerpts may be provided upon reasonable request.

## Footnotes

1 The term ‘women and people with menstrual cycles (WPMC)’ is used in the text to encompass individuals who may menstruate and/or be affected by menstrual cycle-related stereotypes. This terminology is crucial as not all women menstruate, such as trans women or cis women with hormonal disorders, and not all individuals experiencing a menstrual cycle are women, like trans men or non-binary people. We deliberately refrain from using the term ‘menstruators’, common in feminist discussions, as it excludes women who do not (currently) menstruate but still face discrimination related to stereotypes and stigma. Furthermore, our study addresses not only menstruation but the entire menstrual cycle, as well as menopause and perimenopause.

2 Own translation from German.

3 It is worth considering whether the former interviews themselves may have contributed to this shift in the sense of transformative research, which observes and simultaneously intervenes in social realities and practices.

4 We are referring to a newspaper article here, since, as far as we know, no scientific studies have been published on these very recent developments yet.

## Appendix: Practical recommendations for cyclic working environments

The results of our study invite practical interventions that shift the responsibility for change away from WPMC and toward organizations and society. Accordingly, we provide a brief overview of potential organizational and societal interventions. Initiating appropriate forms of engagement can be challenging and complex due to various barriers and perceived fears (see Chapter 4.1). The following recommendations are therefore not exhaustive but should be understood as an initial starting point for further engagement.

### Organization interventions

Ideally, these measures should be introduced as part of a comprehensive strategy, rather than in isolation. The order in which they are implemented depends on organizational needs and capacities, so it does not reflect their relative importance. While some measures may benefit all employees, the recommendations presented here focus specifically on WPMC, as this is the focus of the study.

1. Facilitate menstrual and menopausal management:

a. Sanitary Rooms:

- Provide menstrual products everywhere toilette paper is provided
- Provide clean and well-equipped toilettes

b. Clothing and Furniture:

- Use dark-colored furniture
- Provide or allow breathable workwear

c. Interior Design:

- Provide an appropriate room temperature
- Provide hot water bottles and fans
- Provide institutionalized rest areas

2. Destigmatization and Education:

a. Communicate a culture of trust and safety through, i.e., posters or symbols
b. Educate teams and especially persons in leadership positions about cycle and menopausal health, depending on the team and company size, this could be done via

- Informal exchange rounds between only WPMC or all gender
- Facilitated workshops
- Company wide campaigns

3. Work-time and -place flexibility:

a. Allow workplace and work-time flexibility wherever it is possible
b. Educate people that they are allowed to use this in relation to any issue, thus, also cycle or menopausal related needs
c. Improving the understanding and acceptance of reasons for taking sick leave

4. Cycle awareness:

a. Implement a working culture where wellbeing and health is central to the work organization

- Conscious and scheduled regeneration organization
- Measures of success beyond revenue
- Prioritize emotional and physical wellbeing over profit interest

b. Present these measures as the norm for health, equity and care rather than as a diversity topic

### Societal interventions

As societal interventions can be broader than organizational ones, this collection can be seen as inspiration for creative measures in different parts of society.

1. Cultural Mentalities:

a. Union campaigns following the example of Ireland’s “Stop the Stigma” campaign
b. Educations programs in schools and universities
c. Public information campaign, which could include creative formats, e.g.,

- Printing information about the menstrual cycle on toilette paper in public spaces
- Information on posters in public spaces
- Social Media campaign

2. Political and legal frameworks:

a. Country specific evaluation of current sick leave law and menstrual leave law and adapting it to be more efficient for cyclic and menopausal symptoms
b. Implementation of cyclic, and menopausal health as part of the labor law
